# Polydroxyalkanoates Production from Simulated Food Waste Condensate Using Mixed Microbial Cultures

**DOI:** 10.3390/polym17152042

**Published:** 2025-07-26

**Authors:** Konstantina Filippou, Evaggelia Bouzani, Elianta Kora, Ioanna Ntaikou, Konstantina Papadopoulou, Gerasimos Lyberatos

**Affiliations:** 1 School of Chemical Engineering, National Technical University of Athens, Iroon Potechneiou 9, Zografou, 15780 Athens, Greece; filippoukonstandina@gmail.com (K.F.); bouzani.evangelia@gmail.com (E.B.); lyberatos@chemeng.ntua.gr (G.L.); 2 Institute of Chemical Engineering Sciences (ICE-HT), Stadiou Str., Platani, 26504 Patras, Greece; e.kora@iceht.forth.gr; 3 Department of Environmental Engineering, University of Patras, Seferi 1st, 30100 Agrinio, Greece; 4 Department of Civil Engineering, University of Patras, University Campus, 26500 Patras, Greece

**Keywords:** polyhydroxyalkanoates, condensate, food waste, draw-fill reactors

## Abstract

The growing environmental concerns associated with petroleum-based plastics require the development of sustainable, biodegradable alternatives. Polyhydroxyalkanoates (PHAs), a family of biodegradable bioplastics, offer a promising potential as eco-friendly substitutes due to their renewable origin and favorable degradation properties. This research investigates the use of synthetic condensate, mimicking the liquid fraction from drying and shredding of household food waste, as a viable substrate for PHA production using mixed microbial cultures. Two draw-fill reactors (DFRs) were operated under different feed organic concentrations (2.0 ± 0.5 and 3.8 ± 0.6 g COD/L), maintaining a consistent carbon-to-nitrogen ratio to selectively enrich microorganisms capable of accumulating PHAs through alternating nutrient availability and deficiency. Both reactors achieved efficient organic pollutant removal (>95% soluble COD removal), stable biomass growth, and optimal pH levels. Notably, the reactor with the higher organic load (DFR-2) demonstrated a modest increase in PHA accumulation (19.05 ± 7.18%) compared to the lower-loaded reactor (DFR-1; 15.19 ± 6.00%), alongside significantly enhanced biomass productivity. Polymer characterization revealed the formation of poly(3-hydroxybutyrate-co-3-hydroxyvalerate) (PHBV), influenced by the substrate composition. Microbial community analysis showed an adaptive shift towards Proteobacteria dominance, signifying successful enrichment of effective PHA producers.

## 1. Introduction

The worldwide production of petroleum-based plastics has grown exponentially in just a few decades—from 1.5 million tons in 1950 to 359 million tons in 2018. This has led to a significant increase in the amount of plastic waste generated [1]. It is widely known that the exploitation of petroleum for meeting the current demand of plastic materials poses serious environmental concerns, such as global warming, human health risks, and ecosystem toxicity [2]. Moreover, the inadequate management of plastic waste in landfills results in long-term environmental pollution, requiring urgent research and effective solutions to mitigate its adverse effects on soil organisms, ecosystem safety, and climate change mitigation efforts [3]. Therefore, biobased polymers sourced from sustainable and renewable natural origins are increasingly gaining attention as a promising, environmentally friendly alternative to conventional plastics [4]. Liu et al., for example, have designed a self-healing, recyclable hydrogel for wearable sensors, emphasizing renewable inputs, performance retention, and circular economy strategies, including biodegradability or reusability [5].

Polyhydroxyalkanoates (PHAs) represent a class of bio-polyesters synthesized by various microorganisms, primarily bacteria, as intracellular carbon and energy storage materials. They are accumulated intracellularly in the form of granules when stress conditions of limited bacterial growth and an excess amount of a carbon source exist [6]. The composition and structure of the polymer chains heavily depend on the biocatalyst species and the type and length of the carbon source provided for PHAs synthesis. Depending on the length of the carbon chains in their monomeric units, PHAs are categorized into short-chain-length (scl-PHAs) and medium-chain-length (mcl-PHAs), which consist of 3 to 5 and 6 to 14 carbon atoms, respectively. The scl-PHAs typically exhibit properties similar to polypropylene (PP) and may be mainly used for the production of disposable items and food packaging materials, while mcl-PHAs resemble polyethylene (PET) and polystyrene (PS) in terms of material characteristics, and may be used in various sectors, such as the medical (e.g., implants), pharmaceutical (e.g., biodegradable matrices for drug delivery) and agricultural (e.g., agricultural nets) [7,8,9].

Despite the numerous advantages of utilizing biopolymers, their widespread implementation faces challenges, mainly due to their high production costs. These can be 16 times higher than those of traditional petrochemical plastics [10]. As a result, in recent years, the use of mixed microbial communities (MMCs), capable of PHA production from various waste streams, such as food and agricultural waste, has gained more attention [11]. This is because MMCs can adapt quite well to the complex substrates that may be present in such waste streams. Therefore, utilizing MMCs is considered a more sustainable approach for bioplastics production [6], as they provide significant cost-saving advantages in PHA production, primarily due to their ability to thrive in non-sterile environments [6]. The use of MMCs lowers energy demands and minimizes expenses related to equipment, operation, and maintenance, making the production of PHAs a more feasible approach that can reflect real-world waste valorization setups and align with circular economy and sustainability goals [12].

Approximately 1.3 billion tons of food losses occur globally annually, representing one-third of total food production [13]. The environmental impact of these losses is substantial, with an estimated 3.3 billion tons of CO_2_ equivalent greenhouse gases released annually and a direct economic loss of $750 billion (excluding fish and seafood) [14]. Additionally, in Europe, almost 87.6 million tons of food waste are generated annually. Yet, traditional management solutions often overlook their potential as a valuable resource. Many studies have highlighted the opportunity to utilize food waste to produce high-added-value molecules such as PHAs [15,16,17].

During previous research, an innovative household fermentable waste (HFW) valorization approach was developed and implemented. This approach included the drying/shredding process of the HFW, which results in two streams: a homogenized solid biomass product named Food Residue Biomass (FORBI) and a liquid fraction (condensate), which is collected upon condensation [18]. FORBI is rich in carbon and nitrogen and is an ideal substrate for various anaerobic biological processes for the production of biofuels and electricity [19,20]. The condensate, in general, also has a substantial organic load, mostly volatile fatty acids (VFAs), but has a low nitrogen content. Nonetheless, depending on the drying conditions, the storage time before drying, and the feedstock, the exact characteristics of the condensate may vary [21,22]. As mentioned above, microorganisms generally possess the ability to transform carbon into biopolymers, particularly when subjected to nutrient stress. Therefore, the condensate from food waste drying could serve as an ideal substrate for PHA production.

In this context, the current study focuses on investigating the valorization of condensate from food waste drying as a potential substrate for PHA production. The effect of different organic loadings on the yields and molecular composition of PHAs was studied. To obtain a more homogeneous readily available feedstock, which enables controlled evaluation of the microbial enrichment and PHA production, synthetic condensate was used as the sole carbon source and urea as the organic nitrogen source. The PHA accumulating MMC was developed by subjecting aerobic sludge to alternating nitrogen and carbon limitation. Additionally, the analysis of the microbial community of the PHA-accumulating system was performed. The integration of microbial community profiling under different organic loading conditions provides novel insights into the selection dynamics of PHA-producing consortia.

## 2. Materials and Methods

### 2.1. Setup and Operation of Bioreactors

Two draw and fill reactors (DFR) of working volume 1L each, made of glass, were used. They were operated under non-aseptic conditions at room temperature (27 °C). Agitation was performed using a magnetic stirrer with a stirring rate of 250 rpm, and the carbon to nitrogen ratio was C: N (100 mg COD/mg N). The strategy that was followed for the selection and enrichment of activated sludge in PHA-accumulating bacteria was the alternating cycling between limitation of carbon and nitrogen substrates [23]. In both DFRs, alternating nutrient limitation by sequential supply of C and N allowed the domination of species that can accumulate carbon reserves and overcome nutrient stress. DFR-1 operated for 223 days and DFR-2 for 172 (Table 1). Due to the use of the acclimated biomass from DFR-1 as inoculum, DFR-2 was able to reach stable performance more rapidly.

**Figure 1:**
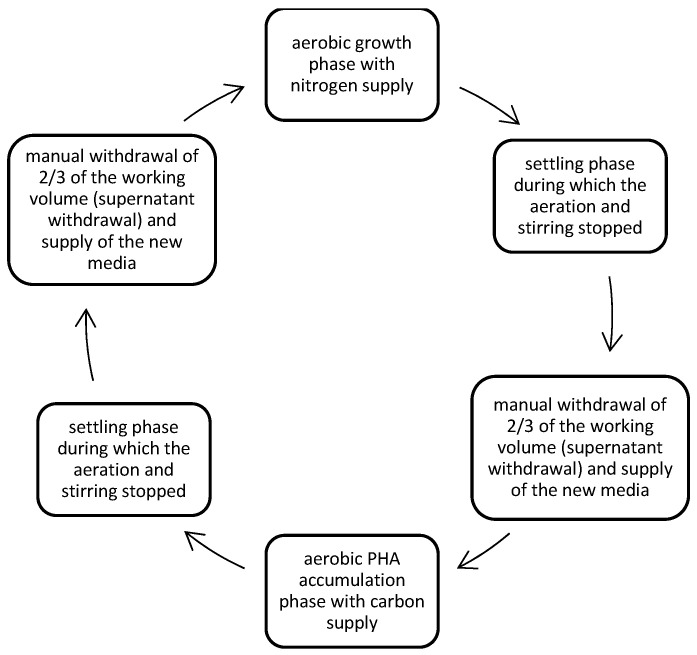
The Operational Mode of the DFRs, which consisted of the sequential cycles of C supply (Accumulation phase of PHAs) and N supply (microbial growth phase).

Specifically, both DFRs’ operations consisted of the sequential phases that are shown in Figure 1. The duration of growth and accumulation phases alternated between two cycles of 47 h and one of 71 h to further suppress the microbial growth of non-PHA producers. The settling phase lasted 55 min, and the withdrawal of the supernatant with the new media supply lasted 5 min. Aeration was conducted with moisturized air at a rate of 2.8 L/min. Following this procedure, PHA-accumulating bacteria could adapt under stress conditions, and bacteria with high PHA-accumulation capacity were able to survive longer than their heterotrophic competitors after several cycles.

### 2.2. Microbial Culture

For the first reactor (DFR-1), an enriched aerobic mixed culture was developed using as inoculum sludge from the recirculation of the secondary clarifier of the Wastewater Treatment Plant of Lykovrisi, Attiki, Greece, during start-up. The start-up was performed using 12.5% *v/v* aerobic sludge and 78.5% *v/v* synthetic media. DFR-2 was inoculated with 30% *v/v* of the enriched MMC from DFR-1.

### 2.3. Feedstock

The synthetic media used in each cycle consisted of either the carbon or nitrogen source, the basal synthetic medium, and a trace element solution. Synthetic condensate was used as carbon source, containing: acetic acid (27% g COD/L), butyric acid (27% g COD/L), propionic acid (6% g COD/L), ethanol (20% g COD/L), glucose (15% g COD/L) and lactic acid (5% g COD/L), with similar composition as the real one (generated from drying and shredding food waste at 98 °C for 9 h and contained: 5% VFAs with an odd number of carbon atoms, 45% VFAs with an even number of carbon atoms, 25% ethanol (EtOH), 24% sugars and 1% lactate. Urea was used as a nitrogen source. The use of synthetic condensate allowed for controlled and reproducible conditions, considered essential for evaluating the effect of organic loading on PHA production. Future studies will incorporate real waste-derived condensates to assess system robustness under more variable conditions.

DFR-1 was operated with a mean feed soluble chemical oxygen demand (sCOD) concentration of 2.0 ± 0.5 g COD/L and 18 ± 0.5 mg/L of nitrogen (N), whereas the feed to DFR-2 had sCOD and N concentrations of 3.8 g/L and 34 mg/L, respectively. The composition of the basal medium used was as follows: 2 g/L MgSO_4_·7H_2_O, 0.05 g/L CaCl_2_·2H_2_O, 12.5 g/L K_2_HPO_4_, 7.5 g/L KH_2_PO_4,_ and 1.0 mL/L of a trace elements solution. The trace elements solution had the following composition: 0.01 g/L CuCl_2_·2H_2_O, 2 g/L FeCl_3_·6H_2_O, 0.03 g/L NaMoO_4_·2H_2_O, 0.02 g/L NiCl_2_·2H_2_O, 1 g/L ZnSO_4_·7H_2_O, 0.2 g/L CoCl_2_·6H_2_O, 0.3 g/L KI, 0.3 g/L H_3_BO_3_, 0.03 g/L MnCl_2_·4H_2_O and 2.5 g/L EDTA.

### 2.4. Analytical Methods

The determination of soluble Chemical Oxygen Demand (sCOD), total suspended solids (TSS), and volatile suspended solids (VSS) was carried out according to Standard Methods for the Examination of Water and Wastewater [24]. The pH was measured using a digital pH-meter (WTW INOLAB PH720, Weilheim, Germany). For the quantification of volatile fatty acids (VFAs), 1 mL of sample was acidified with 30 μL of 20% H_2_SO_4_ and analyzed via a gas chromatograph (SHIMADZU GC-2010 plus, Kyoto, Japan) equipped with a flame ionization detector and a capillary column (Agilent technologies, CA, USA, 30 m × 0.53 mm ID × 1 μm film, HP-FFAP) using an autosampler (SHIMADZU AOC-20 s, Kyoto, Japan). Ethanol, glucose, and lactic acid were measured via High Performance Liquid Chromatography (HPLC) with an Agilent Technologies (Santa Clara, CA, USA)1260 Infinity II HPLC, using the column Agilent Hi-plex H of 300 mm ×  7.7 mm, as described in Kiskira et al., 2023 [25]. Total Nitrogen (TN) was measured with a Shimadzu TN-L analyzer (Shimadzu, Kyoto, Japan; TOC-VCHS and SSM-5000 module). N-NH4+ analysis was carried out using the HI-93733-01 Reagents kit of Hanna Instruments.

The PHAs’ detection method was based on the simultaneous extraction and transesterification of PHAs. The frozen and lyophilized biomass pellets in glass tubes were used for determining the concentration and monomeric composition of the PHAs accumulated in microbial biomass by gas chromatography with flame ionization detection (GC-FID) after acidic methanolysis as described in Oehmen et al. (2005) [26]. Pure poly (3-R-hydroxybutyrate-co-3-R-hydroxyvalerate) (PHBV) copolymer with a 3-R-hydroxyvalerate (3HV) content of 8 mol% from Sigma-Aldrich was used for calibration; thus, the contents of 3-R-hydroxybutyrate (3HB) and 3HV were determined; PHAs were defined as the sum of 3HB and 3HV.

### 2.5. Profiling of the Structure of Microbial Populations

The taxonomic microbial distribution of the inoculum and the changes in the microbial structure of the microbial cultures during the transition from DFR1 to DFR2 operational mode were assessed through Next Generation Sequencing, using samples that were collected upon startup (day 0) and after 108 days for DFR1, and 162 days for DFR2. Total genomic DNA extraction was performed from aliquots of 2 mL well-mixed cultures, using a DNeasy PowerSoil Kit (QIAGEN), Hilden, Germany, whereas PCR amplification procedure, purification, sequencing, and bioinformatics analysis were performed as described in Kora et al., 2022 [8].

### 2.6. Statistical Analysis

Data were tested for normality using the Kolmogorov-Smirnov test. Statistically significant differences of measured and estimated parameters were analyzed using the *t*-test or the non-parametric equivalent, and considering the value *p* ≤ 0.05 as statistically significant.

## 3. Results and Discussion

### 3.1. Comparison of Operational Efficiency of the Reactors

In the present study, synthetic condensate was evaluated as a substrate for the production of PHAs, and the effect of feed organic concentration on PHA content and PHA-accumulating microorganisms’ selection was investigated. Two reactors, DFR-1 and DFR-2, operating at different feed concentrations (2.0 ± 0.5 and 3.8 ± 0.6 g COD/L), were employed. As mentioned in Section 2.1, the carbon-to-nitrogen ratio (C: N) was maintained at 100 mg COD/mg TN, while operational conditions remained the same for both reactors. Various aspects, including sludge properties, substrate utilization, and PHAs synthesis, were monitored.

**Figure 2:**
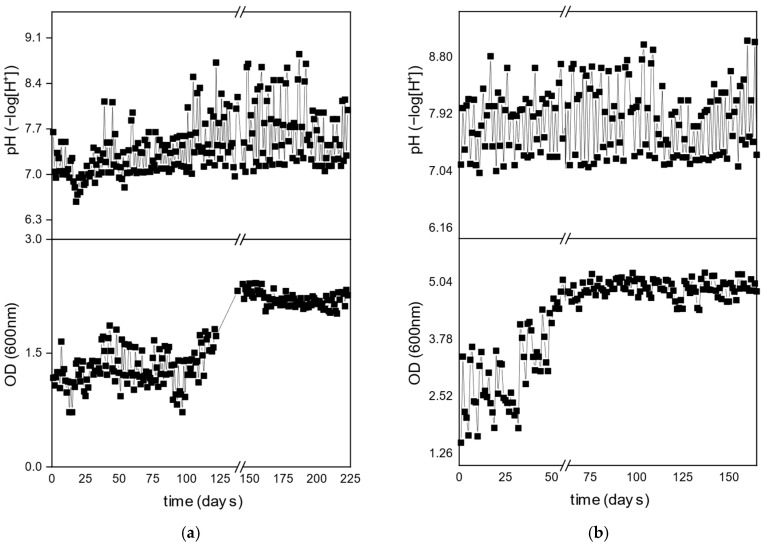
pH and OD_600nm_ for (**a**) DFR-1 and (**b**) DFR-2.

The pH and OD_600nm_ were used for the development of the PHAs accumulating culture in the two reactors throughout the alternating phases of carbon and nitrogen, as shown in Figure 2. The lowest pH and OD_600nm_ values at the start of each cycle correspond to feedstock renewal, during which two-thirds of the reactor volume was withdrawn and replaced with fresh medium. As shown in Figure 2, the pH for DFR-1 and DFR-2, during the N phases, ranged between 7.2–7.8 and 7.4–8.0, respectively. The change in the pH was higher during the C phases (it increased from 7.1 to 8.0 for DFR-1 and from 7.2 to 8.4 for DFR-2) during COD consumption, but the pH remained within ranges that are considered optimal for PHAs production. The microbial biomass increased during the C phases for both reactors, reaching a maximum optical density (OD) value of ~2 for DFR-1and ~2.3 for DFR-2. During the N-phases, it decreased to minimum OD values of ~0.9 and ~1.03, respectively.

**Figure 3:**
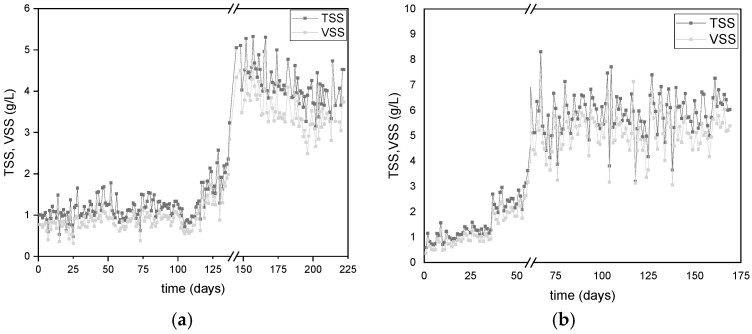
Monitoring of TSS and volatile suspended VSS in (**a**) DFR-1 and (**b**) DFR-2 during alternating carbon-nitrogen phases.

The TSS and VSS values of the microbial cultures are presented in Figure 3. Increases in TSS and VSS indicate microbial biomass growth, while drops at the beginning of each cycle result from the withdrawal of 2/3 of the reactor volume to introduce fresh medium. Overall, there is an increase in microbial biomass for both reactors. By the end of the experimental cycle for DFR-1, the TSS and VSS concentrations reached 3.8 ± 0.6 g/L and 3.2 ± 0.6 g/L, respectively (Figure 3a), while for DFR-2, the values were 6.1 ± 0.5 g/L and 5.2 ± 0.5 g/L (Figure 3b). At the end of the growth phase, a small increase is observed, due to the development of PHA accumulators, consuming the intracellular carbon and the nitrogen provided (urea). In addition, a small solids reduction is observed between the end of each phase and the start of the next, which is due to the removal of 2/3 of the reactor volume to provide the new feed.

**Figure 4:**
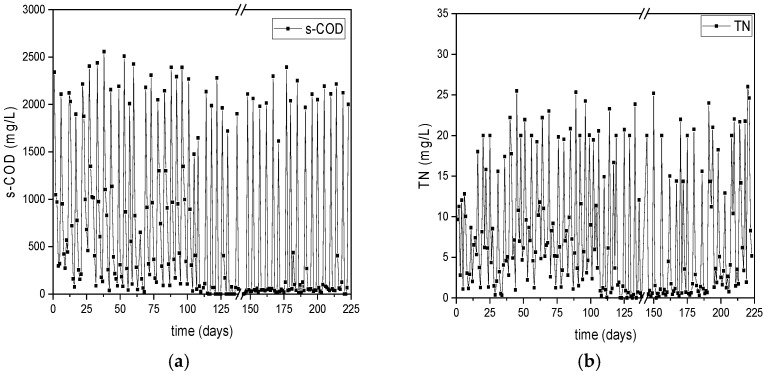
Monitoring the consumption dynamics of (**a**) s-COD and (**b**) TN during alternating carbon and nitrogen cycles in the PHAs-producing reactor DFR-1.

**Figure 5:**
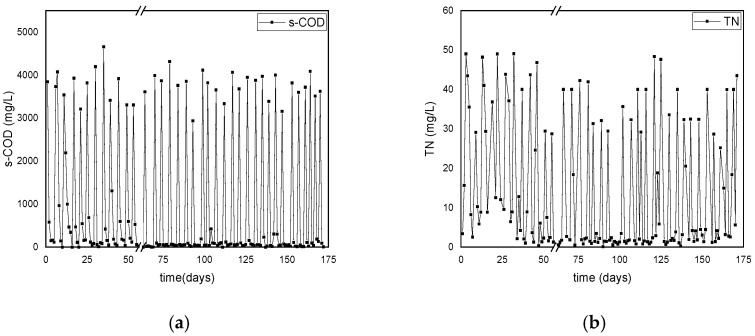
Monitoring of the consumption of (**a**) s-COD and (**b**) TN in the PHAs producing reactor DFR-2.

The consumption of s-COD and TN during the operational periods of the DFR-1 and DFR-2 is illustrated in Figure 4 and Figure 5, respectively. Elevated s-COD values in (a) indicate the onset of the C-cycle, while the following decline reflects carbon consumption and the end of the cycle, during which TN values remain low. Respectively, high TN values in (b) mark the onset of the N-cycle, with its reduction indicating nitrogen consumption and the cycle’s end, during which s-COD is absent.

Regarding the s-COD, both reactors showed a removal efficiency higher than 95% with a similar trend. Specifically, DFR-1 initially displayed a s-COD uptake of approximately 65% until day 110, increasing to nearly 100% within 24 h per carbon cycle thereafter (Figure 4a). On the other hand, DFR-2 demonstrated an average s-COD consumption of 85% over the first two months, achieving complete consumption within 24 h from that point onward (Figure 5a). The absence of carbon during the following growth phase was considered favorable for the performance of the reactors, as it assisted the enrichment of the microbial culture with PHA accumulators. At the onset of each growth phase, TN was 18 ± 5 mg/L for DFR-1 and 34 ± 10 mg/L for DFR-2. Urea introduced during this phase was completely utilized, ensuring nitrogen limitation for subsequent PHA accumulation. Specifically, in DFR-1, urea consumption is observed within the initial 24 h post day 130, as depicted in Figure 4b.

Conversely, in DFR-2, incomplete urea consumption is noted during the 48-h nitrogen phase in the first 30 days, as shown in Figure 5b. This difference may stem from two factors. First, the microbial biomass may not have fully adapted to the new feed concentration, and secondly, the initial microbial cells may have lacked significant intracellularly stored carbon, thus limiting nitrogen uptake. Additionally, until day 35, residual nitrogen remains following the feed switch to carbon, potentially impacting bioplastic accumulation, even though it is fully consumed within 24 h of the carbon cycle. Beyond the 35-day mark, nitrogen is entirely depleted, with consumption within 24 h occurring after day 75, indicative of intracellular carbon presence. Therefore, nitrogen levels at the onset of the carbon phase consistently read as zero, suggesting favorable accumulation conditions.

### 3.2. Comparison of PHAs Yields and Composition

**Figure 6:**
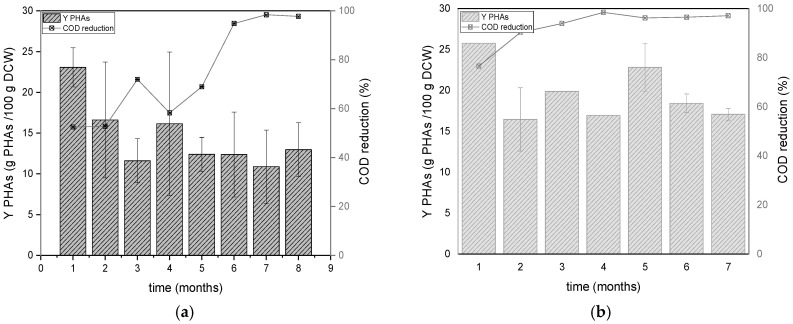
Alterations of the yields of PHAs accumulated at the end of carbon phases and COD reduction during the operation of the DFR-1 (**a**) and DFR-2 (**b**).

In Figure 6, the percentages of PHAs for DFR-1 and DFR-2 and the corresponding COD reduction are shown. Throughout the entire operational period, the average accumulation for DFR-1 was 15.19 ± 6.00%. Regarding DFR-2, the average accumulation percentage is 19.05 ± 7.18%. The increased PHA accumulation observed at higher organic loading rates in DFR-2 can be attributed to the enhanced availability of carbon, which likely facilitated the enrichment of bacterial species with lower metabolic rates. Under limited substrate conditions, such species are typically outcompeted by faster-growing organisms; however, the higher carbon supply in DFR-2 allowed them to establish and persist within the microbial community. These species may not exhibit a competitive advantage under low-carbon conditions but can significantly contribute to PHA synthesis when carbon is abundant [27,28]. Importantly, both DFR-1 and DFR-2 were operated under the same cycle-based regime, with alternating growth and accumulation phases maintained consistently across two 47-h cycles and one extended 71-h cycle. This operational uniformity suggests that the differences observed are due to the feed organic concentration. Moreover, the enrichment under higher carbon availability led to a more diverse and metabolically balanced consortium, including PHA-producing populations well-adapted to the specific operational conditions. This is supported by the higher biomass density (as indicated by elevated TSS and VSS levels in Figure 3).

**Figure 7:**
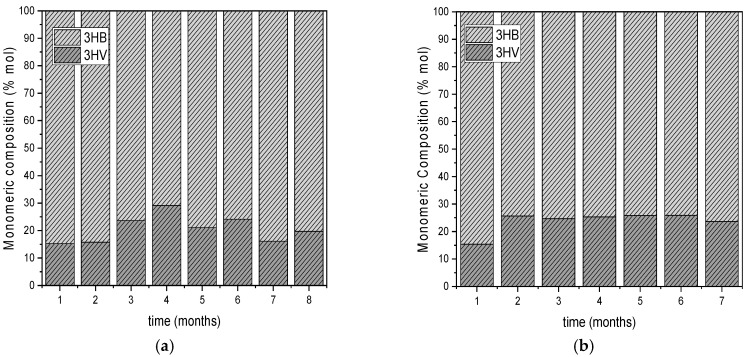
Monomeric Composition of the PHAs accumulated at the end of carbon phases during the operation of the DFR-1 (**a**) and DFR-2 (**b**).

The consistent detection of 3HB and 3HV monomers across all experiments indicates the production of either a copolymer, P(3HBco3HV), or a blend with the homopolymers P3HB and P3HV. Figure 7 shows the monomeric composition of the PHAs accumulated at the end of the carbon phases during the operation of the two reactors. Specifically, in DFR-1, the produced PHAs exhibit an average HB monomer content of 81.41 ± 6.21%, while in DFR-2, this content is 73.79 ± 3.80%. These findings align with existing literature, which suggests that certain carbon sources such as acetic acid, butyric acid, glucose, and lactic acid are metabolized exclusively into P3HB [29,30]. However, when these carbon sources are used alongside valeric acid or propionic acid, as in this study, 3HV is coproduced. Notably, a higher proportion of HV monomer is observed in DFR-2 compared to DFR-1, 26.21 ± 3.80% and 18.59 ± 6.21%, respectively, likely due to the higher concentration of propionic acid in the synthetic feed of DFR-2, which was approximately twice that used in DFR-1. This increase was intended to simulate a higher proportion of odd-chain volatile fatty acids, known to favor HV monomer incorporation during PHA synthesis. As reported in previous studies [8] (Kora et al., 2022), the formation of 3HV in PHBHV copolymer by MMC seems to be primarily influenced by the amount of propionate in the feed. However, the 3HV content in the polymer is not linearly proportional to the propionate concentration. This non-linearity is attributed to intracellular metabolic regulation, specifically the balance between propionyl-CoA and acetyl-CoA, which is governed by enzyme kinetics. Thus, while a higher feed concentration of propionate increases the potential for 3HV formation, as observed in our study, the actual incorporation into the polymer depends on the intracellular metabolic fate of the propionate [8].

In order to compare the two reactors, data were tested for normality using the Kolmogorov-Smirnov test, and the t-test was used to measure and estimate statistically significant differences for normal distributions and their non-parametric equivalent for the non-normal ones (Table 2). Specifically, comparing the two systems, DFR-2 demonstrates a slightly higher accumulation rate, albeit insignificant (*p* > 0.05). The most notable difference lies in the average concentrations of TSS and VSS (*p* < 0.05), with DFR-2 exhibiting approximately 40% higher concentration.

### 3.3. Development of the Mixed Microbial Culture and Evaluation of Its Structure

The operation of DFR1 lasted 8 months, and that of DFR2 7 months. The initial seed seemed to adapt to the limiting conditions in the first reactor, demonstrating a stable behavior in terms of the consumption pattern of the available carbon and nitrogen sources, especially after 4 months of operation. However, since the inoculum of DFR2 was the already adapted microbial consortium of DFR1, it seems that it adapted faster to these conditions. Indeed, the complete limitation of both C and N in the alternating phases was observed, which was maintained throughout the system’s operation.

**Figure 8:**
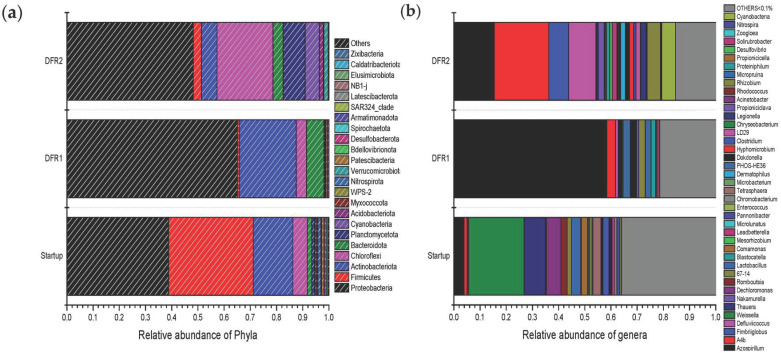
Relative abundance of microbial taxa at the phylum level (**a**) and at the genus level (**b**) during start-up and in the two draw and fill reactors that were used for the development of the mixed microbial culture (MMC) using synthetic condensate as a carbon source.

The composition of the MMC was analyzed via NGS and showed that the distribution of taxa was highly limited compared to the seed culture in both reactors. In Figure 8a, the relative distribution of the bacterial populations at the phylum level is illustrated. As shown, the seed culture (start-up) exhibits a diverse microbial community structure, with Proteobacteria (38.78%) and Firmicutes (32.34%) as the dominant phyla. These groups are typically abundant in activated sludge due to their metabolic versatility and capacity to adapt to fluctuating environmental conditions. Actinobacteriota (15.14%) also play a significant role, likely contributing to substrate degradation [21]. Chloroflexi and Cyanobacteria represented 5.48% and 4.25%, respectively, while Bacteroidota and Planctomycetes each contributed 1.10–1.72%. The initial microbial community composition at the phylum level was characteristic of aerobic sludge [31,32]. However, this distribution changed following the imposition of nutrient limitation. Firmicutes, with a relative abundance of 32.34% in the aerobic sludge, decreased sharply to 0.66% in DFR1, while the population of Proteobacteria increased significantly from 38.78% to 65.13%. Notably, the study by Inoue et al., which investigated aerobic dynamic feeding (ADF) and aerobic dynamic discharge (ADD) processes, also observed a predominance of Proteobacteria [31]. Bacteroidota, which was a minor phylum in the initial seed, increased to 6.67%; this may also be attributed to the adaptation of the respective bacteria to nutrient limitation since it included many species that have an increased PHAs accumulation capacity. Actinobacteriota remain prominent (21.77%), indicating their adaptability and continued role in resource recovery. Conversely, the proportion of Firmicutes plummeted to 0.66%, suggesting their reduced competitiveness under nutrient-limited environments. Thus, the final microbial community composition of the MMC in DFR1 was heavily influenced by the ecological selective pressure applied, leading to an enriched culture for PHAs production.

As mentioned above, the inoculum used in DFR2 originated from DFR1 and, therefore, was already acclimated under stress conditions. However, the increase in the organic load impacted the composition of the MMC. Proteobacteria remained dominant (48.24%) but at a lower relative abundance compared to DFR1 under nutrient stress. The re-emergence of Chloroflexi (21.31%) and an increase in Planctomycetota (8.43%) indicate a shift toward more specialized microbial functions, potentially linked to nutrient cycling and PHA synthesis under the enriched conditions (higher carbon and nitrogen efficiency). More specifically, the relative abundance of Chloroflexi increased from 3.88 to 21.31%. Although there is no direct evidence in the literature for a dedicated metabolic pathway for PHA biosynthesis in Chloroflexi, members of this phylum are indirectly involved in PHA production. It efficiently converts inorganic carbon to key intermediates such as pyruvate. Pyruvate can then feed into metabolic pathways leading to PHA synthesis under specific conditions, especially when carbon sources shift or nutrient limitations occur [33]. The population of cyanobacteria increased noticeably from 0.02% to 5.40%, highlighting their potential as PHAs producers in nitrogen-limited environments. As highly adaptable photoautotrophic organisms, Cyanobacteria have been reported as a promising species for converting CO_2_ into PHAs, which serve as both energy and carbon storage compounds. Moreover, the incorporation of propionic acid as a precursor enables the biosynthesis of PHV, which can occur alongside PHB production, leading to the formation of the PHBV copolymer [34,35,36]. Firmicutes (3.12%) partially recovered, reflecting their adaptation to the increased nutrient availability.

Figure 8b shows the relative distribution of the bacterial populations at the species level, while in Table 3, the ten species with the highest abundance are provided. It is apparent that the progression from the start-up phase to DFR2 demonstrates an evolution toward a microbial community optimized for PHA production. Specifically, upon the start-up of DF1, the microbial community is shown to be quite diverse, with the dominance of Firmicutes (e.g., *Weissella* and *Lactobacillus)* and some Proteobacteria with denitrifying capacity (*Dechloromonas*). Such dominance of fermentative and denitrifying bacteria is expected in activated sludge environments that are geared towards initializing nutrient and organic matter breakdown [37]. Among Proteobacteria, *Azospirillum* emerged as the dominant genus, accounting for 58.40% of the community in DFR1, accompanied by minor contributions from other species. The dominant species is a nitrogen-fixing bacterium that thrives under microaerophilic conditions. Although it is not a traditional PHA producer, studies suggest it can accumulate high quantities of PHB under stress conditions, such as nitrogen or oxygen limitation from various carbon sources [38,39]. This highlights its metabolic versatility and its role as a primary PHA producer in DFR1 [39,40]. Other taxa like *Dokdonella* (2.06%), *Microbulbifer* (3.33%), and *Rhizobium* (2.50%) also belong to Proteobacteria, which are widely studied for PHA production in mixed microbial cultures. These genera are reported to synthesize PHAs at high accumulation levels when subjected to carbon excess and nutrient limitation [11,41]. The low abundance of *Defluviicoccus* (1.08%) and *Micropruina* (2.18%) indicates that glycogen-accumulating organisms (GAOs) were present but not dominant [42]. In conclusion, the dominance of *Azospirillum* highlights a nitrogen-limited system, potentially favoring PHA production, whereas the overall community composition suggests that DFR1 is poised for enhanced PHA synthesis if fed with carbon-rich substrates under nutrient stress.

The microbial composition in DFR2 reflects a stable and diverse ecosystem with a notable presence of both traditional and non-traditional PHA producers, favoring enhanced polymer synthesis. Surprisingly enough, the dominant species was A-4b (20.79%), an unclassified bacterium with limited functional information. However, its significant presence suggests adaptation to reactor conditions and potential involvement in nutrient cycling or polymer storage [43]. *Azospirillum* (15.43%) remained a major contributor, emphasizing its resilience and adaptability to DFR2 conditions, whereas the relative abundance of *Defluviicoccus* drastically increased (10.45%). Indeed, *Defluviicoccus* is a well-documented PHA producer, especially in systems with alternating feast-and-famine conditions, and is a key player in PHA synthesis in mixed microbial cultures. It is classified as a GAO (Glycogen Accumulating Organism), which can accumulate both glycogen and PHA under aerobic conditions when a carbon substrate is available [44,45]. *Fimbriiglobus* (7.59%) and *Rhizobium* (5.18%) are less studied for PHA production but could play supportive roles in the ecosystem. *Rhizobium* strains have been identified as PHA producers, with the ability to synthesize poly(3HB-co-3HV) when provided with glucose as the main carbon source, along with propionate or valerate as supplementary carbon sources [46,47]. Minor taxa like *Leadbetterella* (1.78%) and *Dermatophilus* (1.82%) indicate niche functional roles but are not directly associated with PHA production.

## 4. Conclusions

This study demonstrates the feasibility of utilizing waste-derived carbon and nitrogen sources for the sustainable production of PHAs within a circular economy framework. The successful enrichment of PHA-accumulating microorganisms and the formation of poly(3-hydroxybutyrate-co-3-hydroxyvalerate) (PHBV) in both DFRs confirm that synthetic condensate mimicking food waste liquid fractions can serve as an effective feedstock for biopolymer synthesis. While synthetic condensate was used to ensure reproducibility, future studies will focus on validating the system with real food waste-derived condensates. This will help assess the resilience of the microbial community and the reproducibility of PHA production under more variable, real-world conditions. However, even though food waste is typically rich in indigenous microorganisms, the production process of the condensate involved prolonged thermal treatment (e.g., 175 °C for 10 h), which exerts a pasteurization-like effect. As such, the microbial load in the resulting condensate is expected to be minimal or negligible. This aspect reduces the risk of uncontrolled microbial interference during PHA production and may contribute to a more stable and predictable process, even when using real food waste-derived feedstocks. Microbial community profiling revealed a clear dominance of Proteobacteria, suggesting that targeted enrichment strategies can enhance PHA yields. The high organic pollutant removal efficiencies and stable reactor performance further support the potential of such waste-based substrates in bioplastic production. Notably, the higher organic feed concentration resulted in improved PHA accumulation, indicating that process optimization can enhance yields. While these findings provide strong evidence for the viability of waste-derived carbon sources, further validation using actual food waste condensate will be valuable to confirm scalability and real-world applicability.

## Figures and Tables

**Table 1:** Operational Parameters for the two bioreactors.

Bioreactor	C/N	Feed Concentration (g COD/L)	Days of Operation
DFR-1	100	2 ± 0.5	223
DFR-2	100	3.8 ± 0.6	172

**Table 2:** Summary of the statistical results from the comparison of the two reactors.

	DFR-1	DFR-2	*p*-Value
TSS (g/L)	2.42 ± 1.44	4.34 ± 2.12	0.0000
VSS (g/L)	2.12 ± 1.46	3.84 ± 1.93	
% PHAs	15.19 ± 6.00	19.05 ± 7.18	0.0653
% HB	81.41 ± 6.21	73.79 ± 3.80	0.0236
% HV	18.59 ± 6.21	26.21 ± 3.80	0.0317

**Table 3:** Identification of the strains during start-up and in both DFR reactors to the PHA-forming bacteria cultures by partial 16S rRNA gene.

Start-Up		DFR-1		DFR-2	
*Weissella* (F)	21.30%	*Azospirillum* (P)	58.40%	A-4b (C)	20.79%
2. *Thauera* (P)	8.25%	2. *Dokdonella* (P)	2.06%	2. *Azospirillum* (P)	15.43%
3. *Dechloromonas* (P)	5.52%	3. *Microbulbifer* (P)	3.33%	3. *Defluviicoccus* (P)	10.45%
4. *Lactobacillus* (F)	3.56%	4. *PHOS-HE36* (B)	2.72%	4. *Fimbriiglobus* (PL)	7.59%
5. *Tetrasphaera* (A)	3.14%	5. *Rhizobium* (P)	2.50%	5. *Rhizobium* (P)	5.18%
6. *Comamonas* (P)	2.39%	6. *Micropruina* (A)	2.18%	6. *Nakamurella* (A)	2.31%
7. *Romboutsia* (F)	2.39%	7. *Proteiniphilum* (B)	1.74%	7. *Legionella* (P)	2.19%
8. *Clostridium* (F)	2.33%	8. *Defluviicoccus* (P)	1.08%	8. Dermatophilus (A)	1.82%
9. 67-14 genera (A)	1.62%	9. *Propioniciclava* (A)	0.75%	9. *Leadbetterella* (B)	1.78%
10. Nitrospira (N)	1.18%	10. *Solirubrobacter* (A)	0.72%	10. *Dokdonella* (P)	1.52%
Sum top 10	51.69%		75.49%		67.55%

F, Firmicutes; P, Proteobacteria; A, Actinobacteria; N, Nitrospirota; B, Bacteroidota; C, Chloroflexi; PL, Plancomycota.

## Data Availability

The original contributions presented in the study are included in the article, further inquiries can be directed to the corresponding authors.

## Abbreviations

The following abbreviations are used in this manuscript:

PHA	Polyhydroxyalkanoates
scl-PHAs	short-chain-length Polyhydroxyalkanoates
mcl-PHAs	Medium-chain-length Polyhydroxyalkanoates
HFW	Household Fermentable Waste
FORBI	Food Residue Biomass
VFAs	Volatile Fatty Acids
MMC	Mixed Microbial Culture
DFR	Draw-Fill Reactor
sCOD	Soluble Chemical Oxygen Demand
TSS	Total Suspended Solids
VSS	Volatile Suspended Solids
HPLC	High Performance Liquid Chromatography
TN	Total Nitrogen
GC-FID	Gas Chromatography with flame ionization detection
PHBV	poly (3-R-hydroxybutyrate-co-3-R-hydroxyvalerate)
3HV	3-R-hydroxyvalerate
3HB	3-R-hydroxybutyrate
OL	Organic Load
OD	Optical Density
ADF	Aerobic Dynamic Feeding
ADD	Aerobic Dynamic Discharge
GAO	Glycogen-accumulating Organisms
